# Feasibility of absolute cerebral tissue oxygen saturation during cardiopulmonary resuscitation

**DOI:** 10.1186/cc12546

**Published:** 2013-03-01

**Authors:** Ingrid Meex, Cathy De Deyne, Jo Dens, Simon Scheyltjens, Kevin Lathouwers, Willem Boer, Guy Vundelinckx, René Heylen, Frank Jans

**Affiliations:** 1 Department of Anesthesiology, Intensive Care, Emergency Medicine & Pain Therapy, Ziekenhuis Oost-Limburg, Schiepse Bos 6, 3600 Genk, Belgium; 2 Department of Cardiology, Ziekenhuis Oost-Limburg, Schiepse Bos 6, 3600 Genk, Belgium; 3 Faculty of Medicine and Life Sciences, Hasselt University, Agoralaan, 3590 Diepenbeek, Belgium

## Abstract

**Introduction:**

Current monitoring during cardiopulmonary resuscitation (CPR) is limited to clinical observation of consciousness, breathing pattern and presence of a pulse. At the same time, the adequacy of cerebral oxygenation during CPR is critical for neurological outcome and thus survival. Cerebral oximetry, based on near-infrared spectroscopy (NIRS), provides a measure of brain oxygen saturation. Therefore, we examined the feasibility of using NIRS during CPR.

**Methods:**

Recent technologies (FORE-SIGHT™ and EQUANOX™) enable the monitoring of absolute cerebral tissue oxygen saturation (SctO_2_) values without the need for pre-calibration. We tested both FORE-SIGHT™ (five patients) and EQUANOX Advance™ (nine patients) technologies in the in-hospital as well as the out-of-hospital CPR setting. In this observational study, values were not utilized in any treatment protocol or therapeutic decision. An independent t-test was used for statistical analysis.

**Results:**

Our data demonstrate the feasibility of both technologies to measure cerebral oxygen saturation during CPR. With the continuous, pulseless near-infrared wave analysis of both FORE-SIGHT™ and EQUANOX™ technology, we obtained SctO_2 _values in the absence of spontaneous circulation. Both technologies were able to assess the efficacy of CPR efforts: improved resuscitation efforts (improved quality of chest compressions with switch of caregivers) resulted in higher SctO_2 _values. Until now, the ability of CPR to provide adequate tissue oxygenation was difficult to quantify or to assess clinically due to a lack of specific technology. With both technologies, any change in hemodynamics (for example, ventricular fibrillation) results in a reciprocal change in SctO_2_. In some patients, a sudden drop in SctO_2 _was the first warning sign of reoccurring ventricular fibrillation.

**Conclusions:**

Both the FORE-SIGHT™ and EQUANOX™ technology allow non-invasive monitoring of the cerebral oxygen saturation during CPR. Moreover, changes in SctO_2 _values might be used to monitor the efficacy of CPR efforts.

## Introduction

Monitoring the adequacy of oxygenation and circulation in patients during cardiopulmonary resuscitation (CPR) and advanced life support (ALS) remains a major challenge. Monitoring is limited to clinical observation of consciousness, pulse and breathing pattern [1]. During CPR and ALS, pulse oximetry and non-invasive blood pressure measurement are unreliable. Additionally, an intermittent (every two minutes) rhythm check with electrocardiography (ECG)-electrodes or defibrillator paddles necessitates interruption of chest compressions. Currently, end-tidal CO_2 _measurement is the best technique to monitor adequate circulation; it will increase sharply on return of spontaneous circulation (ROSC). However, end-tidal CO_2 _can only be used in the intubated patient, is dependent on the ventilatory strategy and does not give information on the adequacy of cerebral oxygenation.

An 'ideal' parameter to monitor the adequacy of oxygenation and circulation during CPR and ALS would have the following characteristics: easily (non-invasively) measured, continuous rather than intermittent information, and neither requirement of pulsatile flow nor interruption of chest compressions. Last but not least, it should provide information on the oxygenation of vital organs (heart, kidney, brain).

Cerebral oximetry, based on near-infrared spectroscopy (NIRS) technology, provides information on brain oxygenation and the adequacy of cerebral perfusion [2]. It measures regional cerebral oxygen saturation at the microvascular level (arterioles, venules and capillaries) [3-8]. There have been previous efforts to use cerebral oximetry during CPR with conflicting results [9-11]. Recent developments allow the monitoring of absolute cerebral tissue oxygen saturations (SctO_2_), without the need for calibration (FORE-SIGHT™ technology) [12]. The EQUANOX Advance™ uses similar technology [13], but has the advantage of being portable and as such is more user friendly in the pre-hospital setting. Moreover, the EQUANOX™ technology reveals minimal extracranial contamination compared to other commercially available NIRS technologies [14]. Because of these promising properties (absolute saturation monitoring and transportable monitoring device), we tested the feasibility of both technologies in the CPR setting.

## Materials and methods

In this observational study, data on cerebral tissue oxygen saturation was collected during in- and out-of-hospital cardiac arrest. The SctO_2 _values were not blinded to the attending emergency physicians but were not used in any treatment protocol or therapeutic decision. The study was approved by the Committee for Medical Ethics, Ziekenhuis Oost-Limburg, Genk, Belgium. Requirement for informed patient consent was waived because of the emergency setting.

### Cerebral tissue oxygen saturation

Two emergency physicians, members of the medical emergency intervention team, used non-invasive cerebral monitoring during ALS for patients suffering from cardiac arrest (CA). Initially, a FORE-SIGHT™ monitor (CAS Medical Systems, Branford, CT, USA) was transported to the scene of CA. Due to its weight (9.8 kg), a third person, not taking part in the medical rescue intervention, carried the monitor. On arrival, bilateral NIRS sensors were applied on the patient's forehead. From December 2011 on, the EQUANOX Advance™ monitor (Nonin Medical Inc., Plymouth, MN, US) was used. Since this monitor is light (0.9 kg) and easily transportable, aid from a third person was not necessary. To minimize time delay, only one sensor was applied on the patient's right forehead. With both monitoring devices, SctO_2 _values were electronically collected from arrival until termination of CPR (or until transfer to the emergency unit of the hospital). With both technologies, protective adhesive tape was applied over the sensor(s) in order to minimize possible external light interference.

### Cardiopulmonary resuscitation

Resuscitation procedures were performed in accordance with the Guidelines of the European Resuscitation Council. During ALS, patients were monitored with three-lead ECG and end tidal CO_2 _(ETCO_2)_. Once ROSC had been established, pulse oximetry and non-invasive blood pressure were monitored. In one patient, suffering from in-hospital cardiac arrest, invasive blood pressure monitoring was available during ALS.

### Statistical analysis

Statistical analysis was performed using SPSS V19.0 (SPSS Inc, Chicago, IL, USA). Equal distribution was tested using the Kolmogorov-Smirnov test. Values for cerebral oxygen saturation at each time point were compared using the independent t-test. The results are represented as mean (± standard deviation). A *P*-value below 0.05 was considered statistically significant.

## Results and discussion

In sixteen 16 cardiac arrest patients, NIRS monitoring was applied during CPR (five with FORE-SIGHT™and eleven with EQUANOX Advance™ technology). Relevant differences between the two technologies are listed in Table 1.

**Table 1:** Differences between EQUANOX™ and FORE-SIGHT™ monitors

	EQUANOX™	FORE-SIGHT™
Dimensions (mm)		
height	180	203
width	305	203
depth	130	330
Weight (kg)	0.9	9.8
Time to first value (seconds)	± 10	± 32
Response time (seconds)	4	2
Battery time (hours)	>3	1.5
Extracranial contamination^a^	6.8% ± 6	11.8% ± 5.3
Trouble shoot menu	no	yes

^a^Extracranial contamination was determined as a decrease in cerebral oxygen saturation during inflation of a pneumatic head cuff compared to baseline values [14].

In two patients (EQUANOX™), it was impossible to obtain a value within the first three minutes of monitoring, and no further attempts were made to monitor SctO_2_. Therefore, the results of only 14 patients will be reported.

Three of the five patients monitored with the FORE-SIGHT™ monitor suffered from in-hospital cardiac arrest (IHCA), while the majority of patients (seven out of nine) monitored with the EQUANOX™ monitor suffered from out-of-hospital cardiac arrest (OHCA). Two patients (both OHCA victims) monitored with the FORE-SIGHT™ monitor survived (ROSC >20 minutes). ROSC was observed in four patients (three OHCA and one IHCA) monitored with the EQUANOX™ monitor (Table 2).

**Table 2:** Cerebral oxygen saturation during cardiopulmonary resuscitation

Patient	OH/IH	FS/EQ	ROSC?	Start value SctO_2 _(%)	Highest SctO_2 _(%) during CPR	Highest SctO_2 _(%) during ROSC	SctO_2 _(%) before transport
1	OH	FS	Yes	33	38	73	67
4	OH	FS	Yes	44	53	61	60
6	IH	EQ	Yes	15	58	81	82
11	OH	EQ	Yes	40	41	51	47
13	OH	EQ	Yes	5	10	58	65
14	OH	EQ	yes	37	55	67	46
Mean (± SD)				29 15	43 18	65 11	61 13

**Patient**	**OH/IH**	**FS/EQ**	**ROSC?**	**Startvalue SctO_2 _(%)**	**Highest SctO_2 _(%) during CPR**	**Highest SctO_2 _(%) during ROSC**	**SctO_2 _(%) at end CPR**

2	IH	FS	No	47	45		36
3	IH	FS	No	52	61		35
5	IH	FS	No	51	52		41
7	OH	EQ	No	3	38		0
8	OH	EQ	No	14	61		11
9	IH	EQ	No	7	24		8
10	OH	EQ	No	0	35		25
12	OH	EQ	No	30	42		36
Mean (± SD)				25 22	45 13		24 16

EQ, Equanox Advance technology; FS, Fore-Sight technology; IH, in-hospital cardiac arrest; OH, out-of-hospital cardiac arrest; ROSC, return of spontaneous circulation; SctO_2_, regional cerebral tissue oxygen saturation.

With both technologies, stable NIRS signals and reliable SctO_2 _values were obtained within the first minute of sensor application, except for both patients previously described and excluded from further analysis.

Starting SctO_2 _values (during basic life support) were between 0% and 51%, with a mean value of 27% (± 19) (Table 2). Mean starting SctO_2 _for IHCA was 34% (± 22) whereas mean starting SctO_2 _in OHCA was 23% (± 17), which was not statistically significant (*P *= 0.296). Mean starting SctO_2 _in survivors was 29% (± 15) which was not significantly different from mean SctO_2 _in non-survivors (25% ± 22) (*P *= 0.748). Mean time of CPR before the first SctO_2 _value was monitored was 29 minutes (± 9) in OHCA patients and 16 minutes (± 6) in IHCA patients (Table 3).

**Table 3:** Patient Characteristics

	All patients	OHCA	IHCA
Number	14	9	5
Age, years (± SD)	66 (20)	65 (24)	67 (13)
Male, number	10	6	4
ROSC >20 minutes	6	5	1
Time between CA and first SctO_2 _value, min.^a^	25 (10)	29 (9)	16 (6)

^a^the exact time frame is missing in a number of patients; results are presented as mean (± SD). IHCA, in-hospital cardiac arrest; min., minutes; OHCA, out-of-hospital cardiac arrest; ROSC, return of spontaneous circulation.

The highest SctO_2 _values observed during CPR efforts were between 10% and 61%, with a mean value of 44% (± 15). Highest SctO_2 _values during CPR were neither significantly different between OHCA patients (41% ± 15) and IHCA patients (48% ± 15; *P *= 0,442) nor between survivors (43% ± 18) and non-survivors (45 ± 13; *P *= 0.778) of CA.

ROSC >20 minutes (survivors) was observed in six patients (five OHCA, one IHCA). In the five OHCA patients, highest SctO_2 _values after ROSC were between 51% and 73% with a mean value of 62% (± 8). The only IHCA-survivor showed SctO_2 _values of 81% after ROSC. In these CA-survivors, SctO_2 _values before transfer to the emergency unit were between 46% and 67% with a mean SctO_2 _value of 57% (± 10).

In OHCA patients where any further CPR effort was terminated, SctO_2 _values at the stop of CPR were between 0 and 36%, with a mean SctO_2 _value of 18% (± 16). In IHCA patients, SctO_2 _values were between 8% and 41% (mean 30% ±15) when CPR was stopped, which was not different from the OHCA patients (*P *= 0.311) (Table 2). SctO_2 _values at the end of CPR were significantly lower (*P *= 0.001) in patients not surviving CA compared with those who were transferred to the emergency unit.

**Figure 1:**
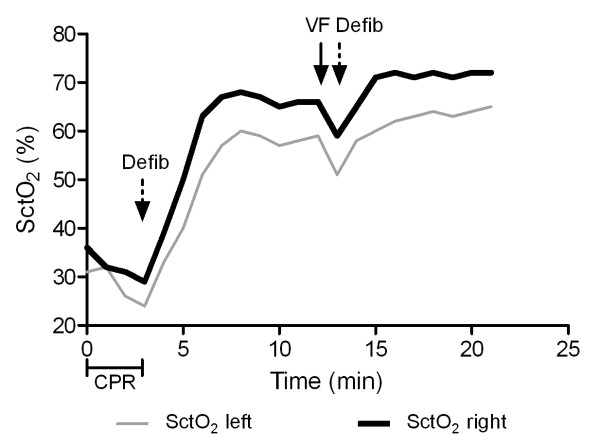
**Cerebral tissue oxygen saturation (SctO2 (%)) (monitored with FORE-SIGHT™ technology) during out-of-hospital cardiac arrest (patient 1)**. After 15 minutes of BLS, first measured SctO_2 _values were 31% and 38% over the left (grey line) and right (black line) fontal region, respectively. Defibrillation (dotted arrow) resulted in ROSC and an immediate increase in SctO_2 _to 60% (left) and 69% (right). During preparation for transport, ventricular fibrillation (arrow) reoccurred and was accompanied with an immediate decrease in SctO_2_. Again, defibrillation (dotted arrow) resulted in sinus rhythm and SctO_2 _values above 65%. BLS, basic life support; CPR, cardiopulmonary resuscitation; Defib, defibrillation; VF, ventricular fibrillation.

**Figure 2:**
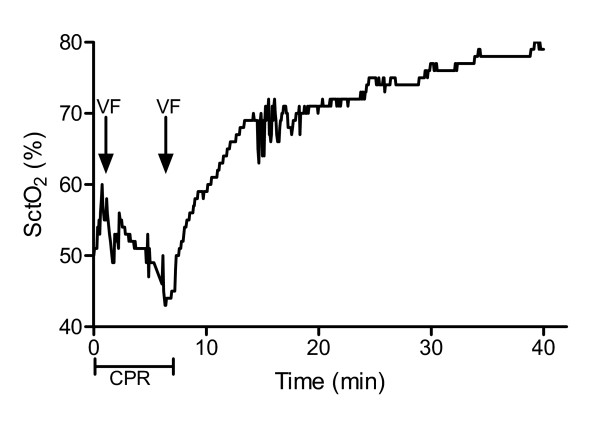
**Cerebral tissue oxygen saturation (SctO_2 _(%)) (monitored with EQUANOX Advance™ technology) in a patient (patient 6) who collapsed at ER entrance**. The sensor was applied after six minutes of CPR and two defibrillation attempts. SctO_2 _started at 50% and increased to 60%, right before ventricular fibrillation reoccurred (arrows). When ROSC was achieved, SctO_2 _increased immediately above 70%. CPR, cardiopulmonary resuscitation; ROSC, return of spontaneous circulation; VF, ventricular fibrillation.

**Figure 3:**
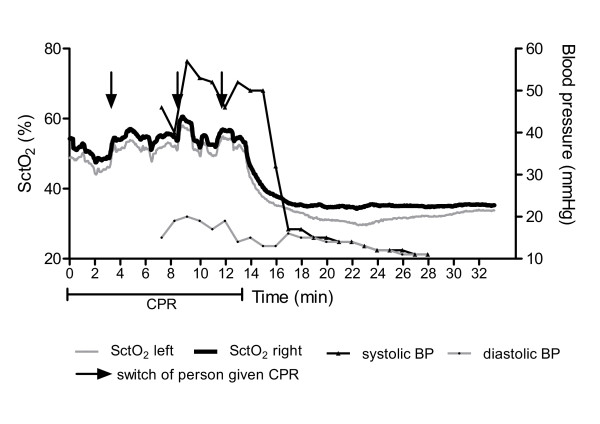
**Cerebral tissue oxygen saturation (SctO_2 _(%)) (monitored with FORE-SIGHT™ technology) and arterial blood pressure (mmHg) in an in-hospital cardiac arrest patient (patient 3)**. After 14 minutes of BLS by a trained caregiver, first measured SctO_2 _values were 48% and 52% over the left (grey line) and right (black line) fontal region, respectively. Switch of person giving CPR (arrows) resulted in increased cerebral oxygen saturations. Parallel fluctuations were observed between SctO_2 _and systolic blood pressure (thin black line). After cessation of CPR, SctO_2 _decreased to values between 30% and 35%. BLS, basic life support; CPR, cardiopulmonary resuscitation.

**Figure 4:**
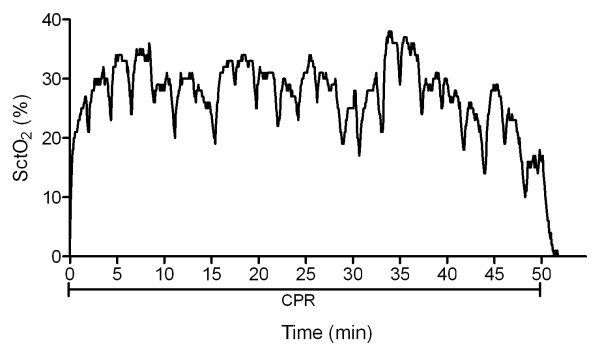
**Cerebral tissue oxygen saturation (SctO_2 _(%)) (monitored with EQUANOX Advance™ technology) during out-of-hospital cardiac arrest (patient 7)**. The starting SctO_2 _value (after 20 minutes of BLS) was 3%, but rose quickly to 21%. The highest measured value during CPR was 38%. A decrease in SctO_2 _was observed during rhythm assessment (every 2 minutes). CPR was stopped 50 minutes after arrival of the medical emergency team, without obtaining ROSC. After termination of CPR, SctO_2 _decreased rapidly. BLS, basic life support; CPR, cardiopulmonary resuscitation; ROSC, return of spontaneous circulation.

As SctO_2 _monitoring during CPR provides continuous information during extremely difficult and rapidly changing conditions, relevant information from SctO_2 _during the CPR efforts is further elucidated by presenting the data of four individual patients (Figures 1, 2, 3, and 4).

This is the first report on the use of the ForeSight™ and EQUANOX Advance™ technology during in- and out-of-hospital CPR. Both technologies use four precise infrared wavelengths to maximize the accuracy of oxyhemoglobin and de-oxyhemoglobin measurement and to enable absolute oxygen saturation monitoring (and not just trend-only monitoring). In a recent comparison of accuracy performance (referring to weighted CO-oximeter reference values), the FORE-SIGHT™ monitor measured cerebral oxygenation most precisely, followed by the EQUANOX Advance™ monitor [15], providing increased confidence in the utilization of this technology in the CPR setting.

Due to the continuous, pulseless wave analysis of both technologies, we were able to monitor the cerebral oxygen saturation in nine OHCA-patients and five IHCA-patients during CPR with two different NIRS devices. The most important difference between the technologies is the respective weight of the monitors. The EQUANOX™ monitoring device is light (0.9 kg) and, therefore, the most suited for use during out-of-hospital CPR.

Newman *et al*. already assessed the feasibility of NIRS to measure cerebral perfusion during OHCA [9]. However, they rarely detected cerebral perfusion or cerebral oxygen saturation using the Invos-3000^® ^technology during CPR. A longer time interval from arrest to initial cerebral oxygen saturation measurements may partially explain the difference between their OHCA report and previous experience during in-hospital CPR [10,11].

Recently, several papers were dedicated to the use of INVOS^®^-NIRS technology in the post-CA area, indicating its potential role in predicting survival and neurological outcome after CA. One of the earliest, although small, OHCA studies demonstrated that all patients surviving for one week achieved a significantly higher median rSO_2 _(regional brain oxygen saturation) during CPR efforts than non-survivors [16]. Also, Ito *et al*. monitored rSO_2 _(INVOS^® ^technology) and noted that any rSO_2 _value below 25%, observed on hospital admission in a post-CA patient without ROSC (despite continued CPR efforts), could be interpreted as a potential indicator of futile resuscitation attempts [17,18]. Kämäräinen *et al*. reported on rSO_2 _(INVOS^® ^technology) and concluded that improving CPR quality did not result in a significant increase in rSO_2 _[19]. It should be noted that they described substantial difficulties in reliable recordings of rSO_2 _data with INVOS 5100C^® ^technology as 59% of their 30-second data had artifacts making quantification of rSO_2 _impossible. Most recently, Parnia *et al*. (using INVOS^®^) concluded that IHCA-patients with ROSC had an rSO_2 _above 30% for >50% of their CPR duration, whereas non-survivors had an rSO_2 _that was below 30% for >50% of their CPR period [20,21]. It therefore stands to reason that cerebral oximetry may have a role in predicting ROSC and the optimization of cerebral oxygenation during cardiac arrest.

In our 14 patients, starting SctO_2 _values were between 0% and 52%. This high variability could be explained by the fact that we measured both IHCA and OHCA patients and that there was a high variability in time from collapse to the first monitored SctO_2 _value in both groups. Furthermore, not all CPR characteristics were available for all patients. Surprisingly, when using the EQUANOX Advance™ monitor, a starting value of 0% SctO_2 _was observed in one patient and an end-of-CPR value of 0% in another patient. Whereas the EQUANOX Advance™ monitor displays these SctO_2 _values of 0%, the lowest SctO_2 _value observed with the FORE-SIGHT™ monitor was around 30%. Due to the proprietary algorithms behind both cerebral oximetry monitors it is impossible to provide any further explanation at present. More data are needed to elucidate the exact significance of these extremely low values and to exclude any possible interference from technical artifacts.

We observed an immediate increase in SctO_2 _after ROSC and found significantly higher SctO_2 _values in patients with permanent ROSC compared to patients in whom no further CPR effort was continued. Furthermore, in four of our patients, new episodes of ventricular fibrillation (Figures 1 and 2) were immediately noted on the SctO_2 _monitoring, as an extra monitoring device indicated this life-threatening situation with urgent need for CPR.

SctO_2 _monitoring provides not only a tool for continuous estimation of cerebral oxygenation during the status of no ROSC, but also the ability to assess the efficacy of CPR efforts. We observed parallel increases in systolic arterial pressure during CPR and in SctO_2 _(Figure 3), illustrating the positive effect of CPR on systolic blood pressure and on cerebral oxygenation. Switch of caregivers during mechanical chest compression, resulted in increased systolic blood pressure and SctO_2 _(Figure 3). The immediate effect of efficient CPR on cerebral oxygenation is illustrated in the rapid decrease in SctO_2 _during rhythm analysis (and consequent interruption of CPR) (Figure 4).

Limitations of this study include the small number of patients, which makes it difficult to reach a conclusion on the exact value of SctO_2 _in CA patients. A second limitation is the fact that we used two different technologies (FORE-SIGHT™ and EQUANOX Advance™). Although both technologies use four wavelengths and display absolute saturation values (without need for calibration), they do use different (proprietary) algorithms to display their calculated SctO_2 _values. The EQUANOX Advance™ oximeter is shown to have less contamination of extracranial vessels compared with the FORE-SIGHT™ oximeter [14], but for both oximeters, accuracy is without doubt acceptable [15]. These differences in algorithms can probably be used to explain the different values displayed in extreme conditions. The major limitation of this study was that it was designed as a pilot feasibility study and that not all patient or CPR characteristics are available. A future, randomized, multicenter study will be initiated on the use of NIRS during CPR with the inclusion of all CPR data following the Utstein CPR data registration. Finally, larger and properly designed studies will have to elucidate the potential role of SctO_2 _monitoring during CPR.

## Conclusions

In conclusion, using FORE-SIGHT™ and EQUANOX™ technology, it is possible to monitor the SctO_2 _during CPR after IHCA and OHCA. Moreover, changes in cerebral oxygen saturation seem to vary with the quality of chest compressions reflecting changes in cerebral oxygenation. Future studies on the prognostic role of cerebral oximetry during CPR are needed.

## Key messages

• NIRS technologies allow measurement of SctO_2 _during CPR.

• These technologies may in the future prove to fulfill the need for better monitoring during CPR.

• SctO_2 _values are the earliest warning signs of impending changes in cerebral oxygenation during/after CPR.

## Abbreviations

ALS: advanced life support; BLS: basic life support; CA: cardiac arrest; CPR: cardiopulmonary resuscitation; ECG: electrocardiography; NIRS: near infrared spectroscopy; IHCA: in-hospital cardiac arrest; OHCA: out-of-hospital cardiac arrest; ROSC: return of spontaneous circulation; SctO_2_: absolute cerebral tissue oxygen saturation; rSO_2_:regional brain oxygen saturation

## Competing interests

The authors declare that they have no competing interests.

## Authors' contributions

IM and CD made substantial contributions to the conception and design of the study, and drafted the manuscript. JD, WB and FJ participated in the design of the study and helped to draft the manuscript. SS and KL were involved in the realization of the study as members of the medical emergency unit. GV and RH were involved in the internal reviewing process. All authors read and approved the final manuscript for publication.
